# Candidates for regulating cytosolic Ca^2+^ influx during inducible aerenchyma formation under low-oxygen conditions

**DOI:** 10.1007/s00299-025-03497-8

**Published:** 2025-05-08

**Authors:** Li Jingxia, Mikio Nakazono, Takaki Yamauchi

**Affiliations:** 1 Bioscience and Biotechnology Center, Nagoya University, Nagoya, Aichi 464-8601 Japan; 2 Graduate School of Bioagricultural Sciences, Nagoya University, Nagoya, Aichi 464-8601 Japan; 3 School of Agriculture and Environment, The University of Western Australia, Perth, WA 6009 Australia

**Keywords:** Aerenchyma formation, Cyclic nucleotide-gated channel (CNGC), Actin microfilaments, Cytosolic Ca^2+^ influx, Ethylene, Reactive oxygen species (ROS)

## Abstract

**The conformational change of CDPKs stimulated by cytosolic Ca**^**2+**^ **influx is essential for activating RBOH-mediated ROS production. CNGCs are promising candidates for regulating cytosolic Ca**^**2+**^ **influx during ROS-dependent aerenchyma formation.**

Oxygen diffusion from the shoot to the root tips through internal gas spaces is essential for plant survival in flooded soils (Colmer and Voesenek 2009). Lysigenous aerenchyma in roots is formed by programmed cell death (PCD) and the lysis of cortical cells (Yamauchi and Nakazono 2022). In rice (*Oryza sativa*), roots constitutively form aerenchyma under aerobic conditions and induce its formation in response to low-oxygen conditions (Yamauchi and Nakazono 2022). The roots of some upland plants, including maize (*Zea mays* ssp. *mays*), also form inducible aerenchyma under low-oxygen conditions (Yamauchi and Nakazono 2022). Constitutive aerenchyma formation in rice roots is regulated by auxin signaling, whereas the inducible aerenchyma formation is stimulated by ethylene and reactive oxygen species (ROS) signaling (Yamauchi and Nakazono 2022).

Ethylene is a key phytohormone that regulates various low-oxygen responses in plants (Bailey-Serres et al. 2025). During inducible aerenchyma formation in rice and maize, ethylene accumulation is increased by enhanced ethylene biosynthesis and reduced gas diffusion in the roots (Yamauchi and Nakazono 2022). Then, the accumulated ethylene stimulates ROS signaling through the transcriptional activation of genes encoding H-clade respiratory burst oxidase homologs (RBOHHs; Rajhi et al. 2011; Yamauchi and Nakazono 2022). Recently, we found that calcium-dependent protein kinases (OsCDPK5 and OsCDPK13) activate the OsRBOHH-mediated ROS production in rice roots (Li et al. 2025). However, how is the cytosolic Ca^2+^ influx, which is required for the conformational change to the active form of CDPK proteins, stimulated during inducible aerenchyma formation is unclear (Fig. 1a).

**Fig. 1:**
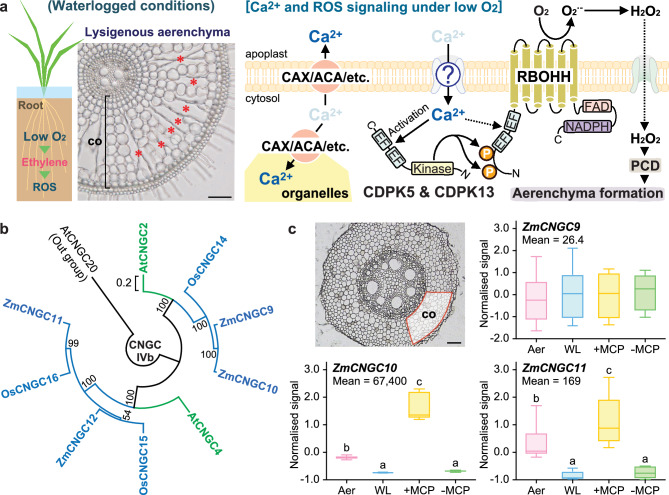
Candidates for regulating cytosolic Ca^2+^ influx during inducible aerenchyma formation. **a** Model of inducible aerenchyma formation in rice roots in response to waterlogged (low-oxygen) conditions. The accumulated ethylene enhances ROS production in the cortex by stimulating the RBOHH expression and cytosolic Ca^2+^ influx from the apoplast. Then, the elevated cytosolic Ca^2+^ activates CDPK5 and CDPK13 and stimulates RBOHH-mediated superoxide radical (O_2_^·−^) production by phosphorylation of N-terminal serine residues. Finally, the accumulated O_2_^·−^ and/or its reduced product hydrogen peroxide (H_2_O_2_) trigger PCD during inducible aerenchyma formation. While Ca^2+^ efflux from the cytosol to the apoplast and/or organelles, which is mediated by CAXs, ACAs, and/or some other proteins, maintain the Ca^2+^ homeostasis, the stimulation of Ca^2+^ influx from the apoplast is required for the transient increase of the cytosolic Ca^2+^ levels. The red asterisks in the image indicate aerenchyma. Scale bar = 100 µm. co; cortex. **b** Phylogenetic analysis of the group IVb CNGC proteins in Arabidopsis, rice, and maize. MEGAX was used to construct the maximum-likelihood phylogenetic tree with bootstrap values calculated based on 100 replicates. The AtCNGC20 (At3G17700) from Arabidopsis was used as the out-group. The scale bar indicates nucleotide substitutions per site. Protein IDs of group IVb CNGCs (Saand et al. 2015); AtCNGC2 (At5G15410), AtCNGC4 (At5G54250), OsCNGC14 (LOC_Os03g55100), OsCNGC15 (LOC_Os01g57370), OsCNGC16 (LOC_Os05g42250), ZmCNGC9 (GRMZM2G074317), ZmCNGC10 (GRMZM5G858887), ZmCNGC11 (GRMZM2G078781), and ZmCNGC12 (GRMZM2G090528). **c** Reanalysis of the mRNA levels of the *CNGC* genes in the cortex of maize (inbred line B73) primary roots using microarray data under accession number GSE22943. Three-day-old aerobically grown maize seedlings were further grown for 12 h under aerobic conditions (Aer), waterlogged conditions (WL), and waterlogged conditions with (+ MCP) or without (-MCP) 12-h pretreatment of 1-MCP (Rajhi et al. 2011). The total RNAs from the cortical cells at the basal part of the roots (1.5–2.0 cm from the root-shoot junction) were isolated by the laser microdissection and used for the microarray analyses (Rajhi et al. 2011). Z-scores normalized the processed signals for each gene. The mean values of all signals for each gene are shown. Different lower-case letters indicate significant differences among different conditions (*P* < 0.05, one-way analysis of variance, followed by Tukey test for multiple comparisons) (*n* = 6). Boxplots show the median (horizontal lines), 25th and 75th percentiles (edges of the boxes), and minimum to maximum (edges of the whiskers). Scale bar = 100 µm. co; cortex

The basal cytosolic Ca^2+^ level is fine-tuned by Ca^2+^ efflux into and influx from the organelles and apoplast (Bose et al. 2011). In *Arabidopsis*, mutants of the genes encoding vacuolar Ca^2+^/H^+^ exchangers (CAXs), *cax1cax3*, fail to reset the elevated Ca^2+^ levels in response to external Ca^2+^ application (Wang et al. 2024). Similar observations have been made in mutants for tonoplast-localized, endoplasmic-reticulum-localized, and plasma-membrane-localized autoinhibited calcium ATPases (ACAs) (Bose et al. 2011). As CAXs and ACAs play a role in Ca^2+^ homeostasis by controlling the Ca^2+^ efflux from the cytosol (Bose et al. 2011), other proteins should trigger the transient cytosolic Ca^2+^ elevation.

Cyclic nucleotide-gated channels (CNGCs) play a key role in the immune response by stimulating cytosolic Ca^2+^ influx in *Arabidopsis* (Tian et al. 2019). Two group VIb CNGCs, AtCNGC2 and AtCNGC4 (Saand et al. 2015), also known as defense, no death 1 (DND1) and DND2, respectively, evoke a robust inward current when co-expressed in *Xenopus* oocytes (Tian et al. 2019). On the other hand, the *cngc2* mutant shows Ca^2+^ hypersensitivity, suggesting that *AtCNGC2* knockout leads to elevated cytosolic Ca^2+^ influx (Chin et al. 2013). Moreover, the autoimmune phenotype in the *cngc2cngc4* double mutant is enhanced compared to that in the *cngc2* or *cngc4* single mutant (Tian et al. 2019). Although AtCNGC2 and AtCNGC4 can form heterotetrameric Ca^2+^ channels (Tian et al. 2019), the absence of AtCNGC2, AtCNGC4, or both may result in the formation of aberrant channels, leading to a substantial Ca^2+^ influx (Chin et al. 2013).

Regulation of PCD during inducible aerenchyma formation resembles the immune response, in which the N-terminal serine residues of OsRBOHH are phosphorylated by OsCDPK5/OsCDPK13 in a Ca^2+^-dependent manner (Li et al. 2025). The expression levels of *OsRBOHH* and its closest homologue in maize (*ZmRBOHH*) in the cortex were strongly induced by low-oxygen conditions, whereas those of *OsCDPK5* and *OsCDPK13* were not (Rajhi et al. 2011; Li et al. 2025). An inhibitor of Ca^2+^ influx from the apoplast prevents inducible aerenchyma formation in rice roots (Yamauchi and Nakazono 2022), suggesting that the CDPK activity is regulated mainly by the cytosolic Ca^2+^ influx. Although the function of CDPKs in inducible aerenchyma formation in maize roots is unknown, an inhibitor of Ca^2+^ influx from the apoplast prevents its formation (Yamauchi and Nakazono 2022).

Previously, we identified the genes whose expression levels were commonly altered in the cortex of maize roots under waterlogged (low-oxygen) conditions when compared with aerobic conditions and waterlogged conditions treated with the ethylene perception inhibitor 1-methylcyclopropene (1-MCP), using laser microdissection combined with microarray analysis (Rajhi et al. 2011). Because *ZmRBOHH* was included in the upregulated genes, its expression in the cortex was stimulated by ethylene under waterlogged conditions. Although no *CDPK* genes were identified, two *CNGC* genes were selected as downregulated (Rajhi et al. 2011). GRMZM5G858887, which was previously described as GRMZM2G074317 owing to a lack of information (Rajhi et al. 2011), has been defined as ZmCNGC10, which belongs to group VIb CNGCs in maize (Saand et al. 2015). Another *CNGC* gene, GRMZM2G078781 (*ZmCNGC11*), also belongs to the group VIb (Saand et al. 2015).

ZmCNGC10 and ZmCNGC11 are close homologues of AtCNGC2 and AtCNGC4, respectively (Fig. 1b), suggesting that ZmCNGC10 and ZmCNGC11 play conserved roles in cytosolic Ca^2+^ influx in maize roots. Because the reduction in *ZmCNGC10* and *ZmCNGC11* expression in the cortex under waterlogged conditions was rather induced by 1-MCP treatment (Fig. 1c), these genes are tightly regulated through ethylene signaling in maize roots (Rajhi et al. 2011). The reduction in *ZmCNGC10* expression under waterlogged conditions was more than 15-fold, and the *ZmCNGC9* expression was lower than that of *ZmCNGC10* (Fig. 1C), suggesting that cortical cells under waterlogged conditions resemble the cells in the *cngc2* mutant in *Arabidopsis* (Chin et al. 2013). Moreover, lower *ZmCNGC11* expression may further stimulate cytosolic Ca^2+^ influx under waterlogged conditions, as in the case of the *cngc2cngc4* double mutant of *Arabidopsis* (Tian et al. 2019).

In conclusion, reducing *CNGC* expression may trigger cytosolic Ca^2+^ influx during inducible aerenchyma formation. *OsCDPK5* and *OsCDPK13* expression is substantial in rice roots, even under aerobic conditions, and their expression levels are not affected by ethylene (Yamauchi and Nakazono 2022; Li et al. 2025). Thus, cytosolic Ca^2+^-triggered conformational changes in OsCDPK5 and OsCDPK13 could be indispensable for activating OsRBOHH-mediated ROS production. The ethylene-dependent reduction in the expression of group IVb *ZmCNGC*s suggests that the elevation of cytosolic Ca^2+^ influx is commonly regulated by group IVb CNGCs in rice and maize during inducible aerenchyma formation. However, rice and maize have two redundant homologues of AtCNGC4, which may contribute to the fine-tuning of PCD during inducible aerenchyma formation. Functional analyses of group IVb CNGC proteins are important to deepen our understanding of plant adaptation to low-oxygen conditions.

## Data Availability

All data supporting the findings of this study are available within the paper. A complete set of microarray data was deposited to the Gene Expression Omnibus (GEO) repository under accession number GSE22943.
